# Clinical Presentation of Acute Pulmonary Embolism in Patients with Coronavirus Disease 2019 (COVID-19)

**DOI:** 10.1155/2020/8855957

**Published:** 2020-11-16

**Authors:** Nonso Osakwe, Douglas Hart

**Affiliations:** ^1^Department of Infectious Disease, New York Presbyterian Hospital, Bronxville, NY, USA; ^2^Department of Cardiology, New York Presbyterian Hospital, Bronxville, NY, USA

## Abstract

The clinical management of severely ill patients with COVID-19-related acute respiratory distress syndrome (ARDS) presents significant challenges. Many COVID-19 patients with ARDS also present with laboratory findings significant for derangement in coagulation function. In this report, we describe acute pulmonary embolism in three patients with COVID-19. We assessed the role of D-dimer assay and anticoagulation treatment in these patients. The aim of this case report is to increase awareness about the use of D-dimer in addition to patient's clinical status for making treatment decision in COVID-19 patients.

## 1. Background

The novel coronavirus that causes COVID-19 disease is the severe acute respiratory syndrome coronavirus 2 (SARS-CoV-2) first identified in late 2019 in Wuhan, China [1]. Many severely ill patients present with acute respiratory distress syndrome (ARDS) with laboratory findings significant for derangement in coagulation function including elevated D-dimer [1, 2]. Previous studies have suggested increased risk of thromboembolism in patients with COVID-19 infection, yet very few case studies exist on this topic [3–5]. We present three patients with COVID-19 disease who were admitted with respiratory failure from pneumonia and were found to have thromboembolism.

## 2. Case 1

**Figure 1:**
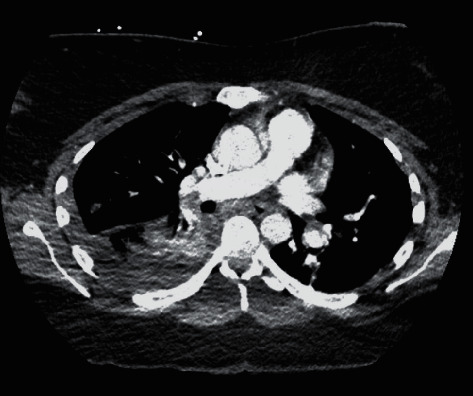
Pulmonary embolism at the level of the bronchus intermedius.

A 26-year-old female without significant medical history and no recent birth control pill use was admitted for shortness of breath, cough, fever, hemoptysis, and pleuritic chest pain after testing positive for the COVID-19 about a week earlier. On admission, the patient had a temperature of 101.5 F, tachycardia of 111 beats/minute, tachypnea with respiratory rate ranging from 19 to 31 breaths per minute, and an oxygen saturation of 95% on room air. She progressively got short of breath and required nasal cannular oxygen at 2-3 liters per minute. Initial electrocardiogram showed sinus tachycardia with ST and T-wave abnormalities. Initial laboratory finding included a white cell count of 16 K/UL (4.5–11.0), negative troponin, negative procalcitonin, and D-dimer elevated at 437 NG/MLDDU (0–243). Chest X-ray revealed multifocal opacities in the lungs bilaterally. The patient was placed on investigational treatment with hydroxychloroquine and azithromycin. Computed tomography (CT) angiogram of the chest was performed due to worsening symptoms; the results showed an acute pulmonary embolism bronchus intermedius with straightening of the interventricular septum suggesting right heart strain (Figure 1). Ultrasound of the lower extremities was negative for deep vein thrombosis. The patient was then placed on anticoagulation with enoxaparin with progressive improvement of symptoms.

## 3. Case 2

**Figure 2:**
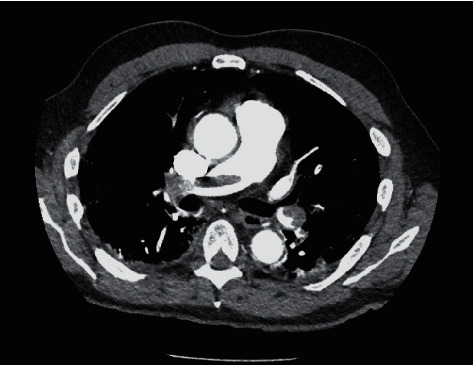
CT angiogram with saddle embolus.

A 65-year-old male without significant medical history was admitted for shortness of breath, cough, and fever. He also tested positive for COVID-19. On admission, he was hypoxic and was started on 4 liters of oxygen via nasal cannula. He was febrile with a temperature of 102 F, respiratory rate in the 20 s, and electrocardiogram with nonspecific T-wave abnormalities. Initial laboratory findings included a white cell count of 5.8 K/UL (4.5–11.0), negative initial troponin, negative procalcitonin, and D-dimer >11000 NG/MLDDU (0–243). Chest X-ray showed patchy mid-to-lower lung predominant airspace opacities concerning for multifocal pneumonia. The patient received investigational hydroxychloroquine and azithromycin; however, respiratory status progressively deteriorated and required nonrebreather oxygen at 15 liters/min. Troponins increased to 1.810 NG/ML (<0.120). Computed tomography (CT) angiogram of the chest was performed (Figure 2) that showed saddle embolus with extension of embolus into the right and left interlobar arteries, the right upper lobe artery, the right middle lobe artery, the left upper lobe artery and segmental arteries of the right upper lobe, the right lower lobe, the left upper lobe, the lingula, and the left lower lobe. The patient was started on heparin drip and subsequently transitioned to apixaban with progressive improvement in symptoms.

## 4. Case 3

**Figure 3:**
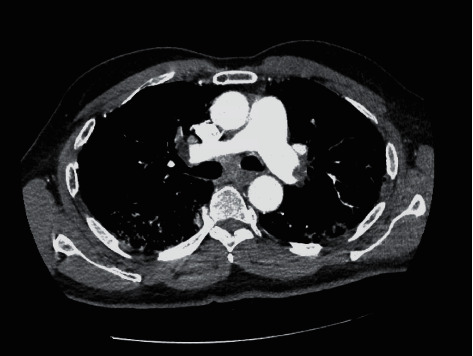
CT angiogram showing pulmonary emboli in the distal right and left pulmonary arteries.

A 66-year-old male with medical history of type 2 diabetes mellitus, hypertension, and hyperlipidemia was admitted for worsening cough, fever, and chills. The patient tested positive for COVID-19. On admission, the temperature was 101.7 F and he was hypoxic requiring a venturi mask FiO2 of 40% to maintain oxygen saturation at 93% and tachypneic with a respiratory rate of 25 breaths per minute. Initial electrocardiogram revealed minimal voltage criteria for left ventricular hypertrophy. Initial laboratory findings included a white cell count of 5.4 K/UL and D-dimer of 152 NG/MLDDU (0–243). The chest radiograph revealed patchy airspace opacities bilateral mid-to-lower lung zones. The patient received investigational treatment with hydroxychloroquine and azithromycin. Respiratory status worsened requiring a nonrebreather mask to maintain oxygen saturation at 90–93%. A repeat D-dimer was 6425 NG/MLDDU (0–243). Computer tomography angiogram due to worsening symptoms and elevated D-dimer revealed pulmonary emboli in the distal right and left pulmonary arteries and segmental and subsegmental bilateral upper lobe pulmonary arteries (Figure 3). The patient was placed on subcutaneous enoxaparin with progressive improvement in symptoms. He was transitioned to apixaban and discharge home.

## 5. Discussion

Wang et al. reported that 71.4% of COVID-19 deaths meet International Society of Thrombosis and Hemostasis (ISTH) criteria for DIC predominantly prothrombotic DIC [2]. Given that D-dimer is a nonspecific marker of inflammation, it is not surprising that the levels increase in COVID-19 cases. Most recently, Chen et al. suggested that patients with COVID-19 pneumonia are at high risk for acute pulmonary embolism when D-dimer remarkably increases [5]; another study suggested that D-dimer values were significantly different between mild and severe disease [1]. In this report, index Case 1 compared to Case 2 had a mildly elevated D-dimer, yet both patients exhibited progressively worsening symptoms. We propose more use of D-dimer elevation as part of treatment decision criteria for acute pulmonary embolism in patients with COVID-19-related ARDS and also encourage a low threshold for further evaluation including CT angiogram especially in patients with worsening or poorly improving clinical status. Closer monitoring of D-dimer in the course of disease may also highlight progression. The clinical question worth exploring is whether higher dose of prophylactic or therapeutic anticoagulation should be administered in patients with COVID-19-related ARDS.
